# The Integration of Top-down and Bottom-up Inputs to the Striatal Cholinergic Interneurons

**DOI:** 10.2174/1570159X22666231115151403

**Published:** 2024-02-29

**Authors:** Yan-Feng Zhang, John N.J. Reynolds

**Affiliations:** 1 Department of Anatomy, Brain Health Research Centre, University of Otago, Dunedin 9054, New Zealand;; 2 Department of Clinical and Biomedical Sciences, University of Exeter Medical School, Hatherly Laboratories, Exeter EX4 4PS, United Kingdom

**Keywords:** Cholinergic interneurons, firing pattern, pauses, top-down input, bottom-up input, integration

## Abstract

**Background:**

Cholinergic interneurons (ChIs) are important for learning and memory. They exhibit a multiphasic excitation-pause-rebound response to reward or sensory cues indicating a reward, believed to gate dopamine-dependent learning. Although ChIs receive extensive top-down inputs from the cortex and bottom-up inputs from the thalamus and midbrain, it is unclear which inputs are involved in the development of ChI multiphasic activity.

**Methods:**

We used a single-unit recording of putative ChIs (pChIs) in response to cortical and visual stimulation to investigate how top-down and bottom-up inputs regulate the firing pattern of ChIs.

**Results:**

We demonstrated that cortical stimulation strongly regulates pChIs, with the maximum firing rate occurring at the peak of the inverted local field potential (iLFP), reflecting maximum cortical stimulation. Pauses in pChIs occurred during the descending phase of iLFP, indicating withdrawal of excitatory cortical input. Visual stimulation induced long pauses in pChIs, but it is unlikely that bottom-up inputs alone induce pauses in behaving animals. Also, the firing pattern of ChIs triggered by visual stimulation did not correlate with the iLFP as it did after cortical stimulation. Top-down and bottom-up inputs independently regulate the firing pattern of ChIs with similar efficacy but notably produce a well-defined pause in ChI firing.

**Conclusion:**

This study provides *in vivo* evidence that the multiphasic ChI response may require both top-down and bottom-up inputs. The findings suggest that the firing pattern of ChIs correlated to the iLFP might be a useful tool for estimating the degree of contribution of top-down and bottom-up inputs in regulating the firing activity of ChIs.

## 1 INTRODUCTION

Striatal cholinergic interneurons (ChIs) are critical for dopamine-dependent learning and act as “gatekeepers” in the basal ganglia [1-6]. *In vivo*, ChIs fire tonically at a rate of 3-10 Hz. When animals receive rewards or sensory cues indicative of reward, most ChIs exhibit a multiphasic excitation-pause-rebound response [7-10]. This response, together with phasic dopamine activity, is critical for shaping corticostriatal long-term plasticity on spiny projection neurons [6]. Furthermore, increasing evidence suggests that ChIs in the striatum are capable of driving axonal dopamine release [11-13] by triggering ectopic action potentials on dopamine axons [14, 15]. Despite their crucial role in dopamine-related learning, the regulation of ChI firing patterns remains unclear [16].

**Fig. (1):**
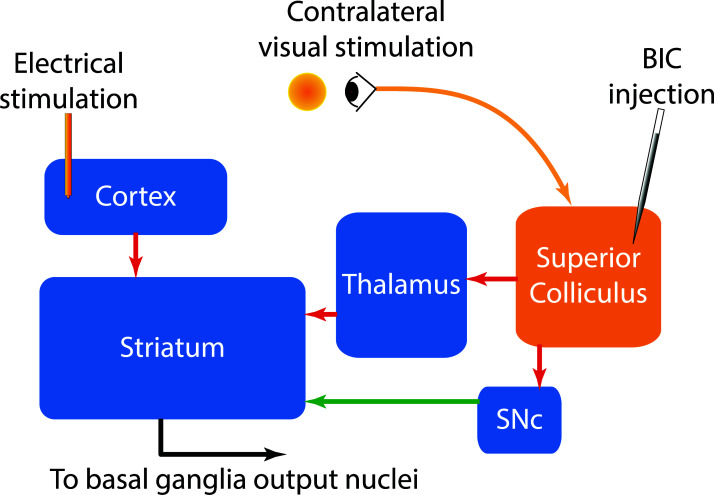
Top-down and bottom-up pathways to the striatum. Electrical stimulation of the cortex can activate the glutamatergic top-down input to the striatum. The injection of BIC, which disinhibits the SC, opens subcortical bottom-up pathways to the striatum. These pathways involve the glutamatergic pathway through the thalamus and dopaminergic inputs from the SNc. The red arrows represent glutamatergic pathways, and the green arrow represents dopaminergic pathways.

ChIs receive intense top-down input from the cortex and bottom-up input from the thalamus and midbrain [12, 17-21]. While inputs from the cortex and thalamus are predominantly excitatory and glutamatergic [12, 20, 22], the input from the midbrain is mainly dopaminergic [23, 24] (Fig. **1**). Additionally, inhibitory and excitatory neurotransmitters co-released from midbrain dopamine neurons [25, 26], and inhibitory inputs from midbrain GABAergic neurons [27] also contribute to regulating ChIs. Although the multiphasic response of ChIs can occur at moderately short latencies (around 200 ms) after a sensory cue indicating a reward, it has been hypothesised that the ChI multiphasic response to visual stimulation may be primarily driven by bottom-up input through visual pathways [28]. This theory is supported by evidence showing that thalamostriatal synapses on ChIs are more proximal than corticostriatal synapses [29], suggesting that the thalamic input may be stronger than the cortical input and, therefore, dominate the firing pattern of ChIs.

In this study, we aimed to investigate the regulation of ChI firing patterns by applying cortical stimulation and visual stimulation individually and in combination. Previous studies have demonstrated that both selective activation of cortical and thalamic pathways can cause pauses in ChIs, as shown in *in vivo* [30] and *ex vivo* [12, 31]. However, electrical or optogenetic activation of these pathways can lead to unphysiological synchronised neuronal activity and may activate neurons not directly involved in visual information processing. Therefore, it remains unclear whether visual input alone can trigger ChI pauses *via* bottom-up input, including the tecto-thalamo-striatal and tecto-nigro-striatal pathways. By employing a physiological meaningful visual stimulation, our results demonstrate that bottom-up input may be too slow to fully account for the short-latency ChI multiphasic response observed in behaving animals. Furthermore, we found that top-down and bottom-up pathways may not share neural circuits in regulating ChI firing patterns, as indicated by the fact that iLFP, a proxy for cortical input, does not represent bottom-up input to ChIs. Moreover, our findings suggest that the top-down input to ChIs may play a similar important role as bottom-up input in regulating ChI firing patterns. Therefore, it is likely that both top-down and bottom-up inputs are necessary to form a multiphasic response in ChIs.

## 2 METHODS

All procedures in this study were conducted in accordance with approvals granted by the University of Otago Animal Ethics Committee. A total of 136 male Long-Evans rats were used for extracellular recording, yielding 17 pChIs.

### 2.1 Surgery

Male Long-Evans rats (250-450 g) were anaesthetised with urethane (1.4-1.9 g/kg i.p.; Biolab Ltd., Auckland, New Zealand). During recording, the level of anaesthesia was monitored by continuous observation of the band-pass filtered electroencephalogram (EEG) signal (0.01 to 500 Hz). Supplementary urethane was administered *via* an intraperitoneal catheter at any sign of EEG desynchronisation, indicating a reduction in the level of anesthesia. The head was fixed in a stereotaxic frame (Narishige, Japan) and the core temperature was maintained above 36°C by a homoeothermic blanket and rectal probe (TR-100, Fine Science Tools). All wounds and pressure points were infiltrated with a long-acting local anaesthetic (Bupivacaine, 0.5%).

For monitoring of the EEG, a hole was drilled in the skull above the left posterior cortex, and a silver wire electrode was placed against the dura overlying the cortex and fixed in place with dental cement. A flap of bone overlying the cortex was removed to provide access to the recording site in the left medial striatum, and a “well” of dental cement was fashioned around the perimeter of the hole. All coordinates are given in millimetres in relation to Bregma and the midline.

### 2.2 Electrical Stimulation

A round piece of skull overlying the right hemisphere (centred AP +2.0 to +2.7 mm and ML -1.6 to -2.0 mm to Bregma) was removed in order to implant a stimulating electrode into the medial agranular motor cortex. A concentric stimulating electrode (Rhodes NEW-100X 10 mm, USA) was implanted in the medial agranular motor cortex to a depth of 1.6 to 2.4 mm. Stimulating electrodes were connected to constant current electrical stimulators (Isolator-10, Axon Instruments Inc.) Stimulus pulses applied to the cortex were biphasic (0.1-0.2 Hz, 0.1 ms, 300 to 990 µA).

### 2.3 Visual Stimulation

Visual stimuli (10 ms duration, 0.2 Hz) were delivered by a white LED (1500 mcd) that was placed 1-2 cm directly in front of the right eye of the animal. The left eye was covered. LED and electrical stimulating electrodes were connected to constant current electrical stimulators (Isolator-10, Axon Instruments Inc.).

### 2.4 Bicuculline Injections

The drug-filled pipettes were lowered to 4.0-4.2 mm from the brain surface into the deep layers of the superior colliculus (AP -6.5/ ML +1.5 mm) and either supported by the IVM micromanipulator or secured with dental cement. Bicuculline (0.01% in saline, 250 nl) was injected into the superior colliculus at a rate of 400 nl/min.

### 2.5 Extracellular Recording

Extracellular single-unit recordings were made using 5-15 MΩ micropipettes. Electrodes were filled with 1 M NaCl solution with 2% neurobiotin (SP1120, Vector). Only stable neurons with wide average spike waveform (>1.1 ms) were included. Recordings were made *via* either a head stage (model HS-2A) connected to an Axoprobe-1A microelectrode amplifier (Axon Instruments Inc California, USA) or a head stage (NL 100 Neurolog) connected to a preamp (NL104), an amplifier (NL106) and a filter (NL125). Signals were amplified, and band-pass filtered within the range of 0.1 to 10,000 Hz. All waveform data were digitised at 50 kHz through an A-D interface (1401 Micro 2, CED, UK), and acquired using SPIKE2 software (v6 or v7, CED).

All the pChIs included in this study showed a tonic firing pattern (Fig. **S1**). While the lower-threshold spike (LTS) interneurons can also exhibit a tonic firing pattern [32], we set criteria to exclude the LTS neurons in this study: 1) Regular firing pattern, *i.e*., no bursty activity [32-34]. 2) Minimum ISI > 8 ms [33]. 3) Fired at both negative and positive phases of the LFP.

### 2.6 Extracellular Recording Experimental Protocol

After a stable single unit recording was obtained from a pChI, cortical stimulation (0.2 Hz), visual stimulation, or paired visual and cortical stimulation were applied. Bicuculline was injected locally into the deep layers of the superior colliculus before each stimulating protocol.

**Fig. (2):**
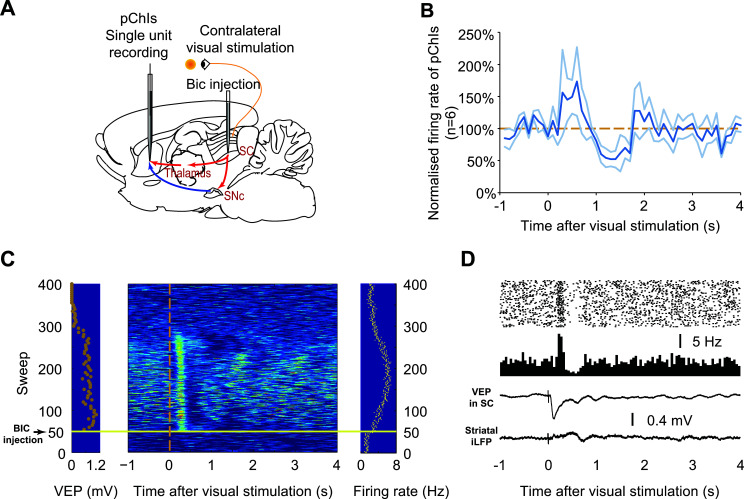
Visual inputs induce varying pauses in pChIs during the effect of BIC-induced dis-inhibition of the SC. (**A**) Contralateral visual stimulation was applied with BIC injected into the SC to regulate pChIs firing pattern. (**B**) Visual stimulation was applied at time 0 s and repeated every 5 s. The average firing pattern (dark blue line) of six pChIs in the first five minutes recording showed an initial excitation around 0.5 s after visual stimulation and a pause between 1s and 2 s following visual stimulation. pChIs then showed a rebound following the “pause.” The Orange dashed line is the average firing rate of the pChI during baseline. Light blue lines are (Mean ± SD). (**C**) A representative pChI recorded during BIC injection and the light flash protocol. Each sweep represents the spikes of the cell over a period of 5 seconds. The BIC ejection and light flash were commenced at the same time (the 50^th^ sweep) (horizontal line). The left panel shows the VEP in the deep layers of the SC during the recording. Each dot in the left panel represents the average of 5 consecutive VEPs. The middle panel is a z-scored raster figure. Warmer colours indirectly represent a higher instantaneous firing rate, and colder colours represent lower instantaneous firing rates. The vertical dashed line indicates the time of the light flash during each sweep. Each dot in the right panel shows the average firing rate of the cell during that sweep. (**D**) *Upper* Raster data shows that visual stimulation alone can induce an excitation-pause-rebound firing pattern of a pChI. The same visual stimulation induces a VEP in the SC (*Middle*) but a very weak fluctuation of the iLFP in the striatum; there is no clear correlation between the iLFP and the firing pattern of the pChI (*lower*).

**Fig. (3):**
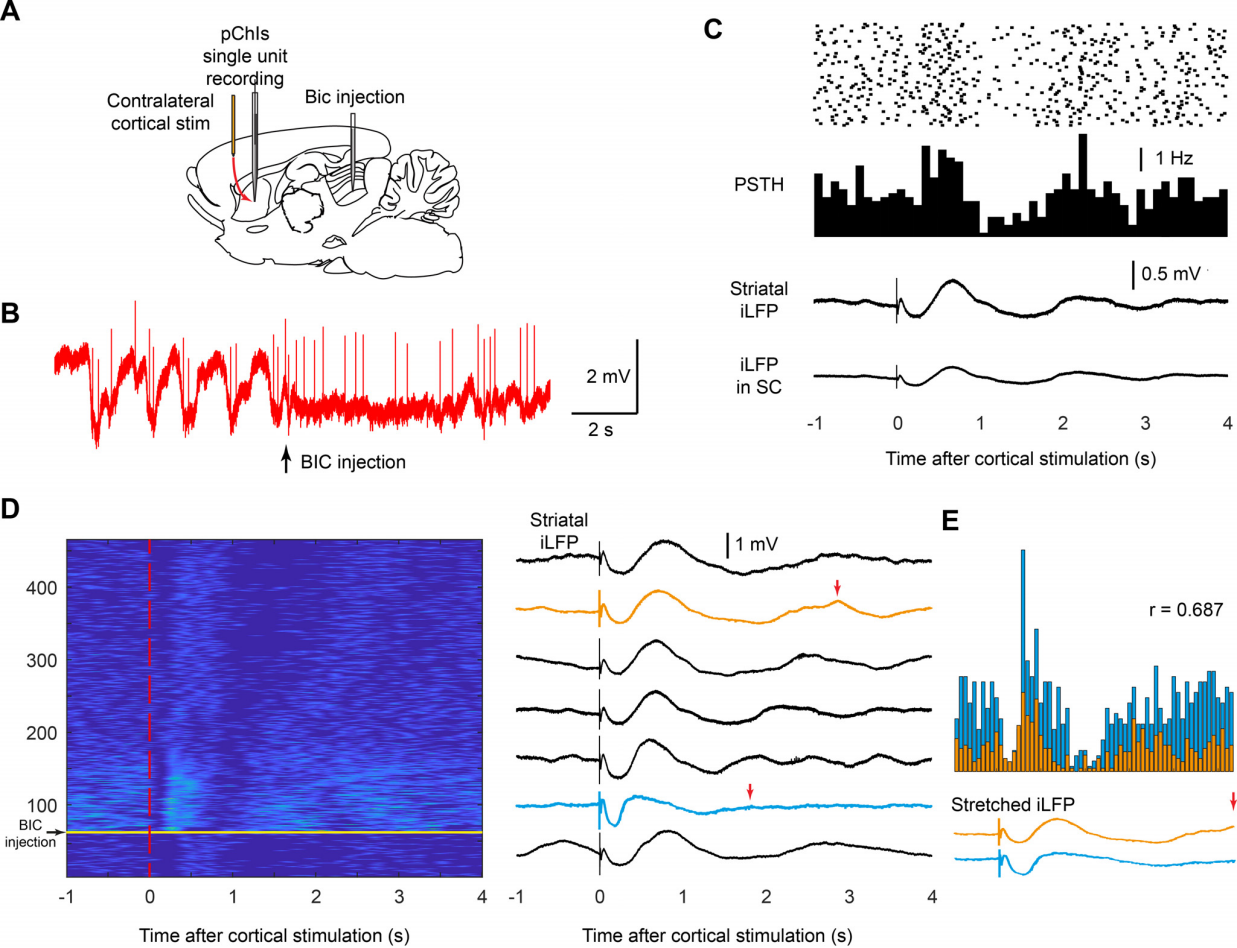
The firing patterns of pChIs were locked to the iLFP regardless of local disinhibition of the SC with BIC. (**A**) Contralateral cortical stimulation was applied with BIC injected into the SC to regulate the pChIs firing pattern. (**B**) Local injection of BIC into the SC desynchronized the striatal LFP caused by urethane anaesthesia. (**C**) Raster data shows that cortical stimulation alone can trigger multiphasic responses in a representative pChI (from n = 13 pChIs) during the BIC effect (*upper*). The iLFP recorded in the striatum and the SC showed a similar pattern due to cortical stimulation (*lower*). (**D**) *Left*: A representative pChI was recorded after BIC injection with the cortical stimulation protocol. Each sweep represents the spikes of the cell over a period of 5 seconds. Warmer colors indirectly represent a higher instantaneous firing rate, and colder colors represent lower instantaneous firing rates in this z-scored raster figure. The vertical dashed line indicates the time of the cortical stimulation during each sweep. *Right*: the averaged iLFPs; each iLFP is an average of 60 sweeps during the ChI recordings shown in the left panel. (**E**) The firing pattern of 60 sweeps from the representative pChI is shown (*upper*) with the associated iLFPs (*lower*) stretched to align the same phases of the iLFP (red arrows in D *left* panel). Despite the recordings being selected right after BIC injection (*blue*) and when the BIC effect was weaker (*orange*), the firing pattern of the ChI locked to the same phases of the iLFP and was highly correlated (Pearson correlation, P = 5.128*10^-10^).

**Fig. (4):**
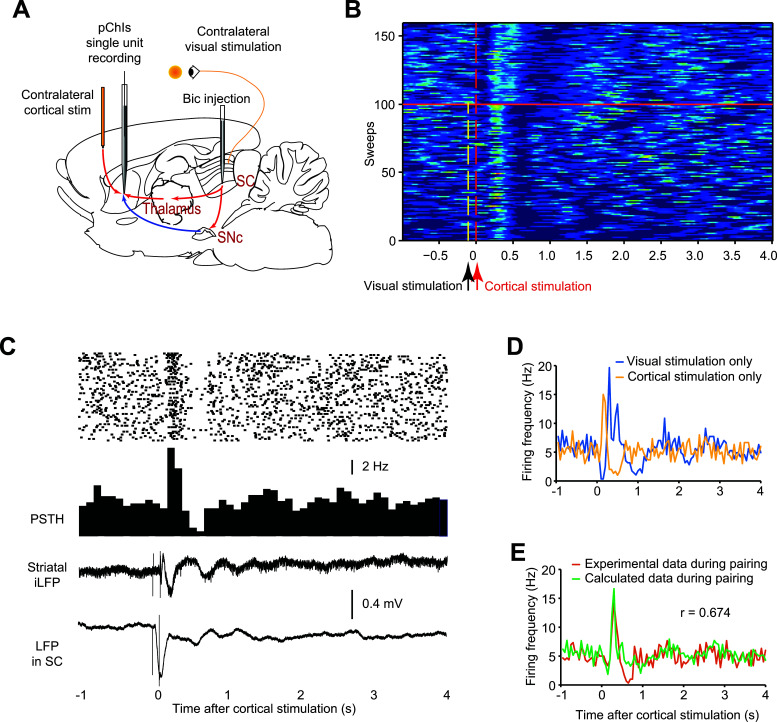
Top-down and bottom-up inputs may contribute equally to the firing pattern of pChIs. (**A**) Paired contralateral cortical and visual stimulation was applied with BIC injected into the SC to regulate pChIs firing pattern. (**B**) The firing pattern of a representative pChI is influenced by both cortical stimulation and visual stimulation under the effect of BIC. Each sweep represents the spikes of the cell over a period of 5 seconds. Warmer colors indirectly represent a higher instantaneous firing rate, and colder colors represent lower instantaneous firing rates in this z-scored raster figure. In the first 100 sweeps, visual stimulation (yellow dashed line) and cortical stimulation (red dashed line) were paired at a 100 ms interval. Visual stimulation was ceased after the 100^th^ sweep (orange line). The firing pattern of the representative ChI changed immediately after the visual stimulation was ceased. (**C**) Raster data shows that paired cortical and visual stimulation alone can trigger multiphasic responses in a representative pChI (from n = 3 pChIs) during the BIC effect (*upper*). The iLFP recorded in the striatum and the SC showed distinct patterns due to cortical stimulation (*lower*). (**D**) Under the effect of BIC, the firing patterns of the same ChI that received visual stimulation (*yellow*) alone and cortical stimulation (*blue*) alone were aligned at the same timing used in the pairing protocol. *i.e*., cortical stimulation is at time 0, and visual stimulation is at time -0.1 s. (**E**) The average of the two firing patterns induced by cortical and visual stimulation alone (*green*) was similar to the real firing pattern obtained during pairing protocol (*orange*), especially the timing and amplitude of the excitation (Pearson correlation, *P* = 1.363 * 10^-6^). However, there is a clearer and more sharply defined pause in firing induced by combined stimulation.

The raster figure and peristimulus histograms (PSTH) of pChIs spikes were plotted using visual stimulation (Figs. **2C** and **D**) or cortical stimulation (Figs. **3C** and **E**, Fig. **4C**) as the triggers.

### 2.7 Data Analyses

Data were analysed offline using SPIKE2 v6 or v7, and MATLAB2020a. Statistical tests on data from single cells as well as on group data were performed in Prism.

## 3 RESULTS

### 3.1 Bottom-up Inputs to pChIs

Contralateral visual stimulation has been shown to drive bottom-up inputs to putative cholinergic interneurons (pChIs) (Fig. **S1**) in the striatum [28]. This results in burst activity of the pChIs, followed by a prolonged afterhyperpolarization (AHP), which leads to a long pause (~1 s) in pChIs firing. It has been proposed that this pause phase may result from burst activity and a subsequent AHP. However, these conclusions were based on a relatively short period of intracellular recording (~5 minutes) of ChIs obtained from extremely low-yield intracellular recordings of ChIs *in vivo*. It is unknown whether these conclusions remain valid with extended recordings or whether visual stimulation can drive a 200 ms long pause as seen in behavioural animals.

To investigate whether the pause in pChIs is directly driven by their burst activity through the AHP, extracellular recordings of pChIs were conducted in urethane-anaesthetised rats to enable longer recordings. Contralateral visual stimulation was applied at 0.2 Hz, and a GABA_A_ antagonist bicuculline (BIC) was locally injected into the superior colliculus (SC) to enable bottom-up inputs to pChIs [6, 35, 36] (Fig. **2A**). The firing pattern of a short period recording (~5 min) of six pChIs was found to be consistent with previous findings, showing an “excitation-pause-rebound” firing pattern where initial excitations were followed by a long pause of about 1-2 s (Fig. **2B**).

However, longer recordings showed a varying firing pattern of pChIs over time after BIC injection. We found that the SC responded to the visual stimulation with an evoked potential (VEP) almost immediately after BIC injection. The size of this VEP then decreased over time and eventually returned to 0 mV at about 250 sweeps of recording after BIC injection (Fig. **2C** *left*), indicating that the BIC effect can last for about 20 minutes. Accordingly, the firing frequency (averaged over each 5s recording epoch) of the representative pChIs initially increased and then returned to pre-BIC levels at about the same time as the VEP disappeared (Fig. **2C** *right*). As shown previously [28, 37], visual stimulation can evoke an excitation phase in the pChIs, which starts from about 250 ms after visual stimulation and lasts for 250 ms. Here, this excitation of pChIs persisted during the whole period of the BIC effect. However, we noticed that the pauses following excitation varied during the BIC effect. In the first 50 sweeps, there was a long pause at 1-2 s after visual stimulation. In the next ~100 sweeps, a faster but shorter pause occurred at about 0.5-0.7s after visual stimulation, and during the last 50 sweeps of the BIC effect, another long and slow pause (1.0-1.8 s after visual stimulation) appeared (Fig. **2C** *middle*). Because the excitation phase of the pChI is consistent during the whole BIC effect, the different pauses we observed were unlikely a direct product of the preceding excitation. The evolution of pauses over time is likely due to changes in BIC disinhibition of the SC after being injected into deep layers of the SC, gradually opening the visual pathway, and the effect of BIC slowly wearing off.

We conducted further investigations to determine whether the pauses observed in pChIs following visual stimulation were caused by prolonged AHPs. If this were the case, pChIs would not fire any action potentials during the pauses. However, we discovered that pChIs did not remain completely silent during the pauses but instead fired at a slower rate *e.g*., (Fig. **2D**). This indicates that the pChI pauses triggered by visual stimulation are unlikely the result of prolonged AHPs. The prolonged AHPs observed in the intracellular recordings may reflect the reduced excitatory input to the pChIs, leading to a decrease in their firing rate.

We then explored whether the firing pattern of pChIs is correlated to the inverted local field potential (iLFP) recorded near the pChIs, where the iLFP serves as a proxy for excitatory input from the cortex [24]. Our earlier study showed that the pause of the ChIs might be induced by the withdrawal of the excitatory input. When electrical cortical stimulation is applied in isolation to urethane anaesthetised rats, the pChI pause occurs during the descending phase of the inverted LFP (iLFP) [24]. However, when visual stimulation was applied, the ascending phase, instead of the descending phase, of the iLFP in the striatum coincided with the time of the pause in pChIs (Fig. **2D**), indicating the bottom-up input to pChIs can modify the firing patterns of the pChIs without influencing the cortical input to the striatum.

### 3.2 Top-down Input to ChIs

We then explored the effect of top-down inputs from the cortex on ChI firing patterns in the presence and absence of BIC injection into SC. When rats are under urethane anaesthesia, earlier works showed cortical stimulation resulted in a multiphasic response in ChIs [18, 30], with pChIs being excited at the peak of the iLFP, which reflects maximal cortical input, and pausing during the descending phase of the iLFP, which indicates the withdrawal of cortical input [24]. However, when we applied visual stimulation here after injecting BIC into the SC, the firing pattern of pChIs did not follow these rules. Instead of pausing during the descending phase of the iLFP, the pChI pause induced by visual stimulation occurred at the rising phase of the iLFP (Fig. **2D**). It is possible that the disinhibition of the SC, which can desynchronise the LFP in anaesthetised rats (Fig. **3**), disrupted how the firing pattern of pChIs followed the local iLFP as described earlier [24]. Therefore, when the SC is locally disinhibited, the firing pattern of pChIs may not follow the previously described rules in all conditions, including when cortical stimulation is applied.

To test this hypothesis, we applied contralateral cortical electrical stimulation at 0.2 Hz after injecting BIC into the SC (Fig. **3**). Compared to ipsilateral stimulation, the contralateral cortical electrical stimulation applied here minimises the chance of directly activating the thalamus *via* corticothalamic pathways. We observed the desynchronisation of the striatal iLFP after local disinhibition of the SC (Fig. **3B**), which is consistent with previous findings [28]. Next, we investigated whether the firing pattern of pChIs still locks to the same phases of the iLFP after contralateral cortical stimulation under the effect of BIC. We found that contralateral cortical stimulation was still able to trigger a multiphasic response in iLFP and pChIs, even after local injection of BIC into SC (Fig. **3C**). The iLFP showed similar but faster multiphasic fluctuations, with each phase being much shorter than before BIC injection (Fig. **3D**). These faster multiphasic fluctuations of iLFP and firing patterns gradually slowed down as the BIC effect wore off (Fig. **3D**). When we examined the correlations between the iLFP fluctuation and ChI firing patterns, we found that the firing pattern of pChIs was still locked to similar phases of the iLFP during the entire BIC effect, indicating that the iLFP still reflected cortical input regardless of the local disinhibition of the SC when only cortical stimulation is applied (Fig. **3E**).

### 3.3 The Integration of Top-down and Bottom-up Inputs

We then asked how pChIs integrate top-down and bottom-up inputs (Fig. **4**). To answer this question, three different stimulation protocols were applied when recording from pChIs: (i) cortical stimulation applied alone to activate top-down input to the ChIs; (ii) visual stimulation applied alone to activate bottom-up inputs; and (iii) the two stimulations paired, with visual stimulation preceding cortical stimulation by 100 ms (Fig. **4A**). The order of the protocols was randomised in the three pChIs where we successfully applied all three protocols. By comparing the response of pChIs to individual and paired stimulations, it was possible to determine which stimulation regimen dominated the firing pattern of pChIs. For an accurate comparison, all three stimulating protocols were applied to the same pChIs, and BIC was injected right before each stimulation protocol.

When the three different stimulating protocols were applied, with the repeated injection of BIC prior to each protocol, we found that pChIs responded to the visual or cortical stimulation similarly to other pChIs that had received only a single protocol of cortical or visual stimulation and a single injection of BIC (see Fig. **4D**). Thus, multiple injections of BIC did not change the way pChIs reacted to cortical or visual stimulations. We then applied the paired stimulation and found that pChIs showed an excitation followed by a brief pause (Fig. **4B**). When the visual stimulation was withdrawn, the firing pattern of the representative pChI immediately changed (Fig. **4B**), suggesting that both stimulations regulated the firing pattern of the pChI together.

To identify whether one of these inputs dominates the firing of pChIs, the firing pattern of the pChIs recorded during cortical stimulation and visual stimulation alone, both under the effect of BIC, were aligned to emulate the pairing protocol. This involved shifting the firing rate recorded with only visual stimulation 100ms forward and then plotting it with the firing pattern recorded only with cortical stimulation (Fig. **4C** & **D**). The comparison could then be made of the effect on the firing pattern of a representative pChI (from n = 3 pChIs) of trials combining the inputs experimentally (Fig. **4E** orange trace) and trials where the two stimulations were applied individually and the averaged effects added mathematically (Fig. **4E** green trace). Notably, the firing patterns of the pChIs from summed individual stimulations were very similar to the experimental results of the combined stimulation in a representative pChI (r = 0.674), especially in terms of the timing (both at 0.3 s after cortical stimulation) and amplitude (16.7 Hz and 14.3 Hz, respectively) of the peak firing rate (Fig. **4E**). However, a much deeper and more-sharply defined pause in firing was induced by the actual combined stimulation. Thus, neither top-down nor bottom-up input dominates the firing pattern of pChIs, but activity from both areas induces a pause response more akin to that observed in behaving animals.

## DISCUSSION

In this study, we investigated the firing pattern of ChIs in the striatum using visual simulation and cortical stimulation. We found that visual stimulation can induce consistent excitation in ChIs but leads to varied pauses when the SC is disinhibited. Thus, pauses in ChIs may not be directly driven by preceding excitations. Additionally, we found that the firing pattern of ChIs was locked to certain phases of the iLFP in the striatum following cortical stimulation regardless of the BIC effect but not in response to visual stimulation. We also found that neither top-down nor bottom-up inputs dominate the firing pattern of ChIs.

The pause observed in ChIs in behaving animals plays a key role in learning [1, 38]. However, the mechanism that induces the pause has been a long-standing mystery in neuroscience. The excitatory thalamic input has long been considered the major input inducing the multiphasic excitation-pause-rebound ChI responses. This hypothesis is supported by evidence such as the lesioning of the thalamic CM-Pf complex in monkeys [39], which prevents the development of ChI pauses, and the fact that thalamostriatal synapses on ChIs are denser and more proximal than corticostriatal synapses [29]. In addition, while ChIs pause after sensory cues in classical conditioning [9, 10, 40], it has been thought that visual and auditory inputs alone are able to drive ChI pauses in classical conditioning.

However, our study found that although visual input can activate tecto-thalamo-striatal and tecto-nigro-striatal pathways to drive a ChI, visual stimulation alone was unable to drive a pause with similar latency and duration as seen in behaving animals. Instead, the visual stimulation drove long-latency pauses (~500 ms, Fig. **1B**-**D**) following the excitation. These pauses are too late compared to the pauses (~200 ms following sensory cues and lasting for ~200 ms) seen in behavioural animals. Therefore, it is likely that other brain areas are also involved in the development of the pause responses in ChIs.

We propose that input from other brain areas, *e.g*. cortex, is also involved in regulating the multiphasic excitation-pause-rebound response of ChIs. Our study provides *in vivo* evidence that cortical input can also powerfully regulate the firing pattern of ChIs, which is consistent with previous *in vivo* studies [6, 18, 30]. Our findings suggest that neither top-down nor bottom-up inputs dominate the firing pattern of ChIs in the striatum, including input through the techno-thalamic-striatal pathway, but together, they produce a powerful pause in firing.

The correlation between the firing pattern of ChIs and the iLFP can be a useful tool to estimate the relative contributions of top-down and bottom-up inputs in regulating the firing pattern of ChIs in real time. Our study found that when cortical stimulation was applied alone, the firing pattern of pChIs was tightly locked to the iLFP, with maximum firing rates occurring at the peak of the iLFP and pauses at the descending phase of the iLFP. Local disinhibition of the SC did not alter the correlation between the firing pattern of pChIs and the iLFP. However, visual stimulation did not produce a similar correlation between the firing pattern of pChIs and the iLFP. Interestingly, when comparing the firing patterns of pChIs recorded with paired cortical and visual stimulations and simulated firing patterns from individual stimulation, we found that the cortical and visual stimulations contributed almost equally to the firing pattern of the ChIs. These results are consistent with our previous report, where visual stimulation was applied after cortical stimulation, although our previous results were obtained from different pChIs due to the difficulty of stable recording from pChIs [6]. Therefore, the degree of locking between the firing pattern of ChIs and the iLFP could be used as an indicator of the relative contributions of top-down and bottom-up inputs to the firing pattern of ChIs. Tighter locking between the firing pattern of ChIs and the iLFP may represent stronger regulation from cortical input, while shifted locking to the iLFP may be used as a proxy for the extent to which bottom-up input regulates the firing patterns of ChIs. Notably, we used bicuculline to disinhibit the SC under urethane anesthesia. Therefore, it is possible that in awake animals, there may be a different degree of deviation between the firing pattern of ChIs and iLFP. A more detailed characterisation of the relationship between iLFP and ChI firing patterns and bottom-up input would need to be made in awake animals to determine the contribution of top-down and bottom-up inputs to ChIs during behaviour. Investigation of how the firing pattern of ChIs is regulated *in vivo* will greatly benefit from more effective recording methods in the future.

Growing evidence suggests that dopamine and ChIs exhibit a complex interplay during behavioural tasks [41, 42]. The discussion of this topic has intensified since the discovery that ChI activity can trigger axonal dopamine release through ectopic action potentials [14, 15]. Our previous study demonstrated that the D2 current from phasic dopamine activity is too slow to elicit a coincident pause in ChIs but that dopamine signals can potentiate the development of ChI pause responses [24]. Here, we further propose that the excitatory input from both top-down and bottom-up pathways may contribute to the formation of ChI multiphasic responses and work in concert to induce effective pauses in ChI firing. In addition to the Kv7-mediated potassium current (I_Kr_) [24], which is triggered by the withdrawal of excitatory inputs to the ChIs, other potential mechanisms, such as prolonged after-hyperpolarisation [18], GABA input from the midbrain [27], and hyperpolarisation-activated potassium current (KIR) [43], may also contribute to the ChI pauses induced by top-down and bottom-up inputs.

## CONCLUSION

In summary, we present evidence that the firing pattern of ChIs can be powerfully regulated by both top-down and bottom-up inputs. The ChI firing pattern is tightly locked to the fluctuations of the iLFP when cortical stimulation, but not visual stimulation, is applied. Our findings suggest that the degree of deviation from how the ChI is locked to iLFP could be an indicator of the contribution of bottom-up input to the firing pattern of ChIs in real time.

## Data Availability

Not applicable.

## LIST OF ABBREVIATIONS

AHP: Afterhyperpolarisation
BIC: Bicuculline
ChI: Cholinergic Interneurons
pChI: Putative Cholinergic Interneurons
iLFP: Inverted Local Field Potential
SC: Superior Colliculus
