# Supplementary material for: The Integration of Top-down and Bottom-up Inputs to the Striatal Cholinergic Interneurons
Source: Curr Neuropharmacol. 2024 Feb 29;22(9):1566–75. doi: 10.2174/1570159X22666231115151403 (PMC11097987; doi:10.2174/1570159X22666231115151403)
Supplement: Supplementary file 1 [file CN-22-1566_SD1.pdf]

Supplementary Material

The Integration of Top-down and Bottom-up Inputs to the Striatal Cholinergic Interneurons

Yan-Feng Zhang[1,2,*] and John N.J. Reynolds[1]

[1]*Department of Anatomy, Brain Health Research Centre, University of Otago, Dunedin 9054, New Zealand;* [2]*Department of Clinical and Biomedical Sciences, University of Exeter Medical School, Hatherly Laboratories, Exeter EX4 4PS, United Kingdom*

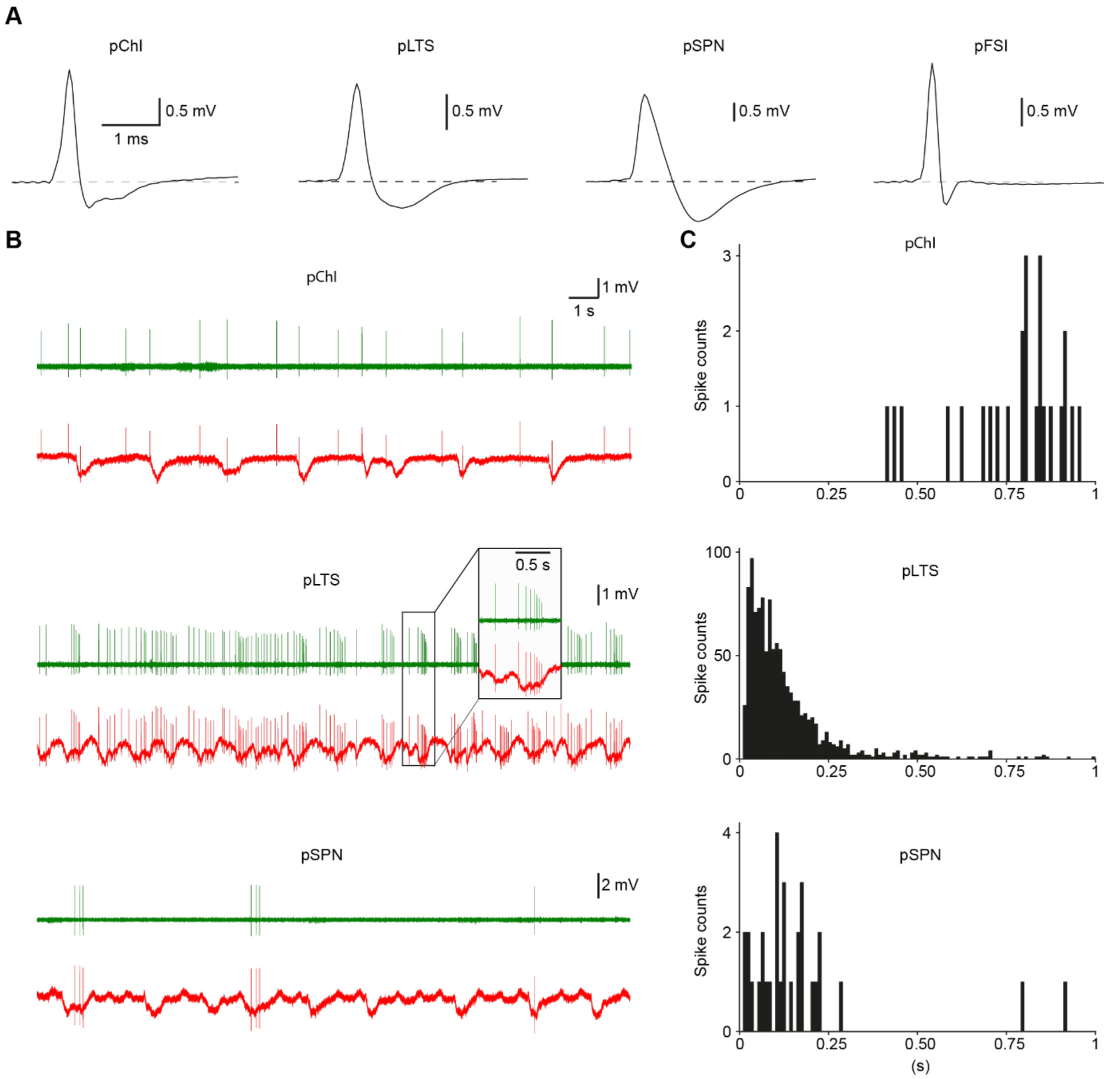

Fig. S1. Identification of pChIs. **A**) The average spike waveform of pChIs, putative low-threshold spike neurons (pLTS), and putative spiny projection neurons (pSPNs) is longer than 1 ms. Putative fast spiking interneurons (pFSI) have much shorter average spike waveforms, which are shorter than 1 ms. **B**) Examples of single-unit recordings (*red*, raw recordings, *green*, high-pass filtered traces) of pChI, pLTS, and pSPN demonstrate that pChIs fire tonically, while pLTS and pSPN fire in bursts. **C**) Only neurons with a minimum inter-spike interval (ISI) higher than 0.1 s were classified as pChI. See the methods section for detailed criteria.

1875-6190/24 $65.00+.00 **© 2024 Bentham Science Publishers**
